# Myeloid-derived suppressor cells in laryngeal squamous cell carcinoma: an underexplored immunosuppressive axis and strategic target for overcoming checkpoint inhibitor resistance

**DOI:** 10.3389/fimmu.2026.1931000

**Published:** 2026-08-17

**Authors:** Guan-Jiang Huang, Xue-Sen Yang, Pei-Shan Li, Zhi-Jun Fan, Biao-Qing Lu

**Affiliations:** 1 Department of Otorhinolaryngology Head and Neck Surgery, Zhongshan Hospital of Traditional Chinese Medicine, Affiliated to Guangzhou University of Chinese Medicine, Zhongshan, Guangdong, China; 2 The Tenth Clinical Medical College of Guangzhou University of Chinese Medicine, Zhongshan, Guangdong, China

**Keywords:** immune checkpoint inhibitors, immunotherapy resistance, laryngeal squamous cell carcinoma, myeloid-derived suppressor cells, tumor microenvironment

## Abstract

Laryngeal squamous cell carcinoma (LSCC) shows a persistently low objective response rate to immune checkpoint inhibitors (ICIs) targeting the programmed death-1 (PD-1) pathway. Its immunosuppressive tumor microenvironment (TME), enriched in tumor-associated macrophages, immunosuppressive tumor-associated neutrophils (TANs), and regulatory T cells, is a principal driver of ICI failure. Yet one myeloid population central to ICI resistance across many malignancies has received no dedicated attention in this disease. Myeloid-derived suppressor cells (MDSCs) are heterogeneous, pathologically activated immature myeloid progenitors that suppress CD8+ T cell and natural killer cell cytotoxicity, promote regulatory T cell expansion, and sustain PD-1 blockade resistance through redundant mechanisms. In LSCC, tumor-derived G-CSF and GM-CSF activate the PI3K-AKT pathway in infiltrating neutrophils and upregulate PD-L1, and this same cytokine axis is an established upstream driver of MDSC differentiation and mobilization. The predominantly elderly, male, HPV-negative, tobacco-exposed patient profile further favors MDSC accumulation through aging-associated myeloid bias, senescence-associated secretory phenotype signaling, and chronic inflammation. Evidence support MDSCs as a clinically tractable immunosuppressive node in LSCC. These are the LSCC tumor secretome, the shared transcriptional landscape of polymorphonuclear MDSCs (PMN-MDSCs) and immunosuppressive TANs, and the clinical validation of circulating LOX-1+ PMN-MDSCs as predictors of ICI resistance in head and neck squamous cell carcinoma (HNSCC). Drawing on this evidence, we outline a mechanistic rationale connecting LSCC biology to MDSC expansion and argue that combining MDSC-targeting strategies with PD-1 blockade merits dedicated clinical investigation in this disease.

## 1 Introduction

Laryngeal squamous cell carcinoma (LSCC) accounts for approximately 100,000 deaths worldwide each year and represents one of the most squamous cell carcinoma of the head and neck (HNSCC) (1). In locally advanced disease, multimodal strategies combining surgery, radiotherapy, and platinum-based chemotherapy have constituted the standard of care for decades. Yet 5-year overall survival in stage III and IV disease remains poor, and functional consequences such as total laryngectomy substantially impair quality of life (1). Immune checkpoint inhibitors (ICIs) targeting the programmed death-1 (PD-1) pathway transformed the treatment of recurrent and metastatic HNSCC. On the basis of improved overall survival in pivotal randomized trials, pembrolizumab achieved regulatory approval in both first-line and second-line settings (2, 3). Despite this advance, objective response rates with pembrolizumab in unselected HNSCC populations remain approximately 17 to 23 percent, and the majority of patients experience primary resistance or early progression (2, 3). This persistent therapeutic ceiling reflects the complexity of the immunosuppressive tumor microenvironment (TME) in HPV-negative tumors, of which LSCC is a canonical example.

Recent studies have clarified the cellular composition of the LSCC TME. Single-cell transcriptomic analyses of metastatic LSCC revealed an immunosuppressive state dominated by regulatory T cells, dysfunctional plasma cells, early-stage macrophages, and abundant neutrophil infiltration at tumor margins (4). A key mechanistic study demonstrated that LSCC tumor cells secrete elevated G-CSF and GM-CSF, which activate the PI3K-AKT signaling pathway in tumor-associated neutrophils (TANs). The result is a pool of aged, immunosuppressive CXCR4+ TANs that directly impair CD8+ T cell function *in vitro* and *in vivo* (5). GM-CSF secretion by LSCC tumor cells further drives PD-L1 upregulation on tumor-infiltrating neutrophils, and GM-CSF neutralization reverses this phenotype in experimental models (6). ANXA3-rich exosomes from LSCC-associated macrophages promote M2-like polarization through AKT-GSK3β-β-catenin signaling and suppress ferroptosis in cancer cells to facilitate lymphatic metastasis (7). Metabolic reprogramming, including alterations in glycolysis, lipid metabolism, and amino acid catabolism, further shapes the immunosuppressive landscape of LSCC (8).

One myeloid population central to ICI resistance in other solid tumors has received no dedicated scientific attention in LSCC. Myeloid-derived suppressor cells (MDSCs) are immature, pathologically activated myeloid progenitors that expand in response to tumor-derived cytokines and mediate potent immunosuppression through multiple complementary mechanisms (9, 10). In multiple tumor types including HNSCC, circulating and intratumoral MDSCs correlate with advanced disease stage, lymph node involvement, and ICI resistance (11, 12). The absence of LSCC-specific MDSC characterization from the research literature is both a scientific gap and a missed therapeutic opportunity.

**Figure 1:**
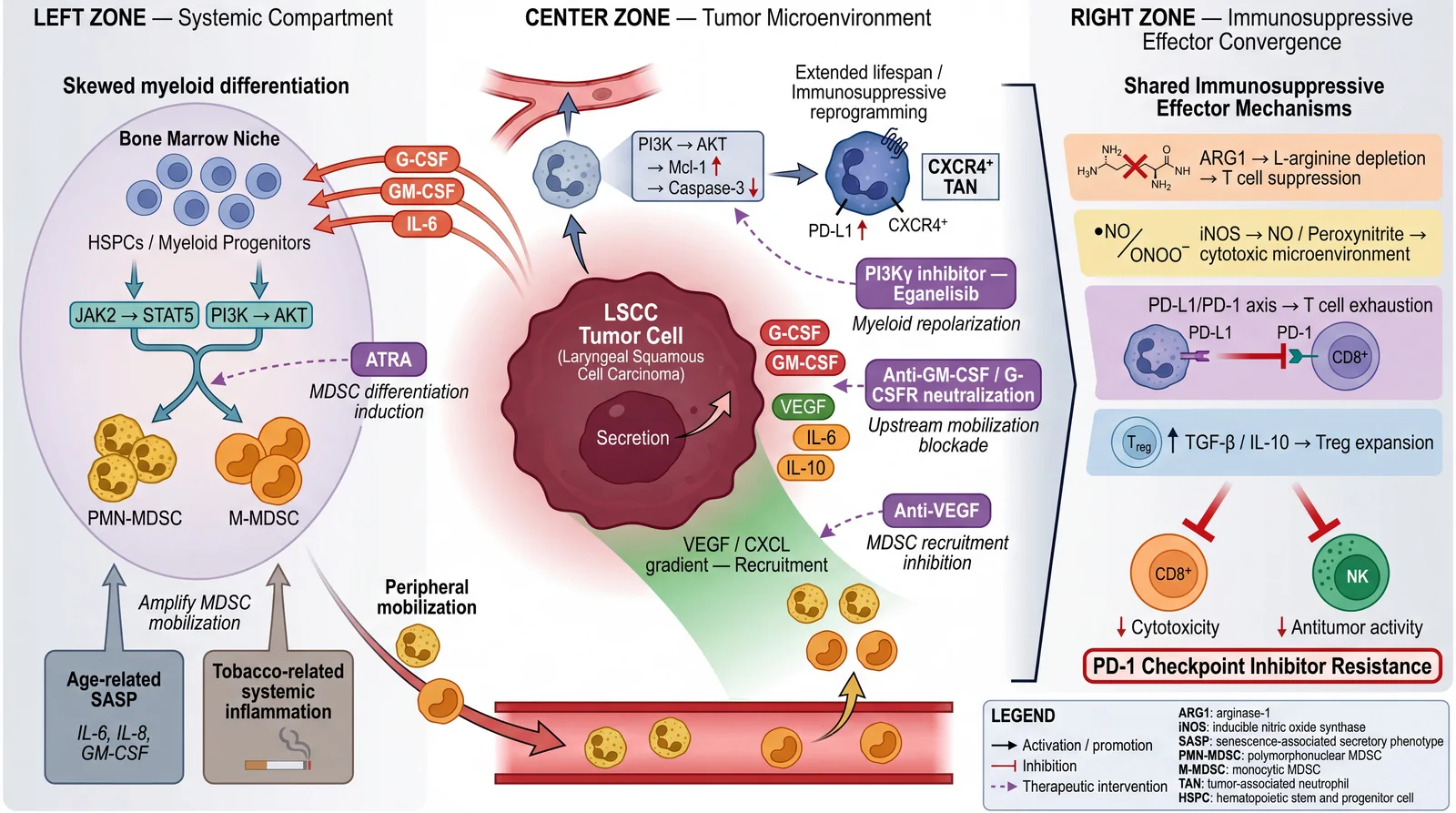
Proposed model of G-CSF/GM-CSF-driven myeloid immunosuppression in the LSCC tumor microenvironment and mechanistic convergence with MDSC biology. LSCC tumor cells secrete elevated G-CSF, GM-CSF, VEGF, IL-6, and IL-10. These factors drive myeloid immunosuppression through two interconnected arms. In the first arm, G-CSF and GM-CSF activate the PI3K-AKT signaling pathway in infiltrating neutrophils, upregulate Mcl-1, suppress Caspase-3-mediated apoptosis, and drive PD-L1 surface expression, generating immunosuppressive CXCR4+ tumor-associated neutrophils (TANs) with pathologically extended lifespan. In the second arm, the same cytokines act on bone marrow hematopoietic progenitors through JAK2-STAT5 and PI3K-AKT to divert differentiation toward immature MDSCs (PMN-MDSCs and M-MDSCs), which are mobilized to the peripheral circulation and recruited to the tumor site by VEGF and CXCL chemokine gradients. Both TANs and MDSCs converge on shared immunosuppressive effector mechanisms: ARG1-mediated L-arginine depletion, iNOS-driven nitric oxide and peroxynitrite production, PD-L1/PD-1 axis-mediated T cell exhaustion, and TGF-β/IL-10-driven Treg expansion. This myeloid-dominant immunosuppressive TME impairs CD8+ T cell and NK cell antitumor cytotoxicity and constitutes a primary mechanism of PD-1 checkpoint inhibitor resistance. Age-related SASP cytokines (IL-6, IL-8, GM-CSF) and tobacco-related systemic inflammation amplify MDSC mobilization from the systemic compartment, compounding tumor-derived myeloid programming. Therapeutic nodes are annotated: ATRA (MDSC differentiation), PI3Kγ inhibitor eganelisib (myeloid repolarization), GM-CSF/G-CSFR neutralization (upstream mobilization blockade), and anti-VEGF (MDSC recruitment inhibition). ARG1, arginase-1; iNOS, inducible nitric oxide synthase; SASP, senescence-associated secretory phenotype; PMN-MDSC, polymorphonuclear MDSC; M-MDSC, monocytic MDSC.

In this Perspective, we develop three arguments (**Figure 1**). First, the established G-CSF and GM-CSF secretory axis in LSCC constitutes a shared upstream driver of MDSC expansion that operates through machinery common to TAN immunosuppressive programming. Second, LSCC-specific biological and demographic features amplify systemic MDSC accumulation. Third, MDSCs constitute a treatable immunosuppressive node whose targeting is warranted on both mechanistic and clinical grounds.

## 2 MDSC classification and core immunosuppressive mechanisms

MDSCs are broadly divided into two principal subsets based on their morphological and phenotypic resemblance to mature myeloid lineages. In humans, polymorphonuclear MDSCs (PMN-MDSCs) carry the CD11b+CD14-CD15+LOX-1+ phenotype, while monocytic MDSCs (M-MDSCs) are defined by CD11b+CD14+HLA-DR-CD15- surface expression (9). International consensus recommendations address how to distinguish bona fide MDSCs from their morphologically similar but non-suppressive neutrophil and monocyte counterparts. The definitive criterion is a demonstrated capacity to suppress autologous T cell proliferation in standardized functional co-culture assays (9). This operational criterion is important in the context of LSCC, where the biological overlap between PMN-MDSCs and immunosuppressive TANs demands rigorous functional validation before claiming MDSC identity.

MDSCs suppress immune responses through several complementary mechanisms. Arginase-1 (ARG1), expressed by both MDSC subsets, depletes L-arginine from the TME, impairing CD3 zeta-chain expression on T cells and blocking T cell receptor-mediated proliferation (10, 13). Inducible nitric oxide synthase (iNOS) generates nitric oxide that, combined with superoxide, produces peroxynitrite, a reactive nitrogen species that nitrates T cell receptor complexes and renders antigen-specific T cells functionally anergic (13). Reactive oxygen species generated through NADPH oxidase activity further oxidize key T cell signaling molecules and accelerate functional exhaustion (13). PD-L1 surface expression on MDSCs provides an additional contact-dependent suppressive signal to PD-1-expressing T cells, creating a redundant checkpoint signal that anti-PD-1 monotherapy only partially neutralizes (13, 14). Through TGF-β and IL-10 secretion, MDSCs promote Treg expansion, suppress NK cell cytotoxicity, and impair dendritic cell antigen presentation, which together reinforce the immunosuppressive microenvironment. Metabolic reprogramming through fatty acid oxidation sustains MDSC survival and function under the nutrient-depleted conditions of the TME. In parallel, IDO-mediated tryptophan depletion and ARG1-driven arginine depletion restrain T cell activation and effector cytokine production. Recent work has shown that reversal of MDSC metabolic programming can restore antitumor T cell function in immunosuppressive TME settings, broadening the therapeutic options for MDSC targeting beyond depletion alone (14).

## 3 The MDSC landscape in HNSCC and the LSCC-specific gap

The immunosuppressive role of MDSCs in HNSCC has been established in recent years, yet LSCC has never been examined as a biologically distinct entity in this literature. Single-cell transcriptomic analyses of HNSCC have demonstrated that MDSCs constitute a functionally significant fraction of the tumor-infiltrating immune compartment. Within this compartment, they establish an immunosuppressive microenvironment through ARG1 upregulation, PD-L1 expression, and downstream Treg induction (11, 12). Within the HNSCC TME, tumor cells recruit MDSCs through secretion of IL-10, TGF-β, GM-CSF, and VEGF, which establish conditions for sustained myeloid expansion (15, 16). A recent study revealed an important dimension of myeloid immunosuppression in HNSCC. It showed that intratumoral bacteria recruit polymorphonuclear myeloid cells, including PMN-MDSCs, to the TME. This recruitment establishes immunosuppression and inversely correlates with T cell infiltration and ICI response across multiple independent cohorts (17).

A multicenter study established the clinical relevance of PMN-MDSCs to HNSCC ICI resistance. In patients with recurrent and metastatic HNSCC undergoing pembrolizumab-based therapy, circulating LOX-1+ PMN-MDSCs independently predicted ICI resistance and inferior survival (18). High pre-treatment LOX-1+ PMN-MDSC levels correlated with significantly worse overall survival and progression-free survival. The same analysis demonstrated that M-MDSCs, which produce lower ARG1 levels in HNSCC settings, did not independently predict outcomes, identifying PMN-MDSCs as the dominant immunosuppressive MDSC subset in this disease (18). Because LOX-1+ PMN-MDSCs are quantifiable by standard peripheral blood flow cytometry, they represent readily applicable non-invasive biomarkers for ICI response prediction and treatment monitoring.

Despite this accumulating evidence in HNSCC broadly, no dedicated study has characterized the MDSC compartment in LSCC as a distinct entity. HPV-positive tumors display markedly higher CD8+ T cell infiltration, stronger activation of immune signaling pathways, and a more immunogenic TME architecture compared with HPV-negative counterparts (19). Because LSCC is almost universally HPV-negative, it falls into the opposing immunophenotypic category, one defined by a myeloid-dominant, T cell-excluded TME. In this setting, MDSC-mediated suppression of the residual antitumor T cell activity carries the greatest clinical consequence. Subsuming LSCC within heterogeneous HNSCC analyses has obscured this distinction and contributed to the neglect of MDSC biology in LSCC. Future research programs must approach LSCC as an independent entity with disease-specific immunological characteristics requiring dedicated MDSC characterization.

## 4 Tumor-derived G-CSF and GM-CSF as shared upstream drivers of myeloid immunosuppression in LSCC

The mechanistic link between established LSCC tumor biology and the probable expansion of MDSCs in this disease rests on the established roles of G-CSF and GM-CSF. LSCC tumor cells secrete elevated levels of both cytokines, which activate the PI3K-AKT signaling pathway in TANs. This activation upregulates Mcl-1, suppresses Caspase-3-mediated apoptosis, and pathologically extends TAN lifespan. The resulting pool of immunosuppressive CXCR4+ TANs impairs CD8+ T cell function both *in vitro* and *in vivo* (5). GM-CSF further drives PD-L1 surface upregulation on LSCC-infiltrating neutrophils, a phenotypic switch reversible by GM-CSF neutralization in experimental models (6). These observations establish LSCC tumor cells as active producers of myeloid-programming cytokines operating through the PI3K-AKT axis.

At the bone marrow level, the same G-CSF and GM-CSF signals drive MDSC differentiation from hematopoietic stem cell progenitors. Under tumor-induced pathological conditions, high-concentration GM-CSF activates JAK2-STAT5 and PI3K-AKT signaling, diverting hematopoietic stem cell differentiation away from mature dendritic cells and macrophages and toward immature, immunosuppressive MDSCs (20). G-CSF drives the expansion of granulocyte-monocyte progenitors and their release into the peripheral circulation as PMN-MDSC precursors. These findings provide molecular evidence that G-CSF exposure generates myeloid cells with PMN-MDSC transcriptional characteristics (21). Subsequent single-cell RNA sequencing confirmed that human circulating PMN-MDSCs and TANs from different cancer types share transcriptomic cell clusters. This finding supports the view that they represent related myeloid cell states arising from common cytokine-driven programming (22).

This mechanistic convergence is directly applicable to LSCC. The G-CSF and GM-CSF secretory program established in LSCC tumor cells operates through the PI3K-AKT signaling axis to generate immunosuppressive TANs with PMN-MDSC-like functional characteristics. We propose that the aged, CXCR4+, immunosuppressive TANs accumulating in LSCC under G-CSF and GM-CSF stimulation represent, at minimum, the intratumoral component of the PMN-MDSC continuum in this disease. Rigorously distinguishing PMN-MDSCs from immunosuppressive TANs in LSCC will require a combined approach. This work has not yet been undertaken and remains the most scientifically treatable gap in the field.

Generalizability was confirmed across other solid tumors. The mechanistic link proposed here between G-CSF/GM-CSF signaling and MDSC-driven ICI resistance is not unique to LSCC but reflects a convergent, pan-cancer axis increasingly documented across malignancies. In hepatocellular carcinoma, the tumor-intrinsic transcription factor ETV5 was recently shown to directly bind JAK2 and activate STAT3-dependent transcription of CCL2, thereby driving PMN-MDSC expansion, tumor infiltration, and immune checkpoint inhibitor resistance (23). This mechanistic architecture closely parallels the PI3K-AKT/JAK2-STAT5-driven TAN-to-PMN-MDSC continuum proposed for LSCC. In colorectal cancer, granulocytic MDSCs recruited via GM-CSF, IL-8, CXCR2, and CCL2 signaling confer resistance to checkpoint blockade, particularly in the mismatch-repair-proficient subset that mirrors the immunologically “cold”, myeloid-dominant phenotype of HPV-negative LSCC (24). In pancreatic ductal adenocarcinoma, a tumor type long regarded as paradigmatic for GM-CSF-driven myeloid immunosuppression, pharmacological disruption of this axis with the HDAC inhibitor entinostat converted a subset of checkpoint-refractory tumors into responders in a phase II trial (25). This cross-cancer evidence base indicates that the G-CSF/GM-CSF-MDSC-ICI resistance axis constitutes a generalizable, druggable mechanism rather than an LSCC-specific speculation, reinforcing the biological plausibility of its dedicated investigation in this disease.

## 5 LSCC-specific biological and demographic amplifiers of MDSC accumulation

Three features intrinsic to the LSCC patient population create conditions particularly permissive for MDSC accumulation beyond what is attributable to the tumor secretome alone. These features distinguish LSCC from other HNSCC subsites in important ways.

### 5.1 Aging and the senescence-associated secretory phenotype

LSCC predominantly affects elderly males, with median ages at diagnosis typically exceeding 60 years in major clinical series. Aging is accompanied by immunosenescence, progressive myeloid-biased hematopoiesis, and the senescence-associated secretory phenotype (SASP). SASP-associated cytokines, particularly IL-6, IL-8, and GM-CSF, are chronically elevated in aged individuals and function as potent MDSC mobilization and activation signals (26, 27). Age-related increases in IL-1β and TNF-α from dysfunctional bone marrow stromal cells further drive myeloid-biased differentiation and chronic low-grade inflammation, expanding the MDSC progenitor compartment at the expense of lymphoid output (28). Studies have documented a statistically significant increase in circulating MDSC frequency in elderly individuals compared with younger adults. MDSCs from aged donors also exhibit enhanced T cell suppressive capacity, stronger polarization toward type 2 and regulatory immune phenotypes, and altered metabolic function (26, 27, 29). A single-cell transcriptomics study defined the developmental trajectory of MDSCs in aged mice, identifying distinct transcriptional programs not present in young controls that include upregulation of metabolic reprogramming and immune checkpoint genes (30). In the HNSCC clinical setting, a phase 2 trial recently validated immunosenescence as a mechanism of ICI resistance. Poor responders showed higher expression of immunosenescence-related genes in T and B cell subsets and lower levels of CCR7+ CD4+ naive T cells (31). These aging-associated changes add a systemic route of MDSC amplification to the tumor-derived, cytokine-driven expansion described above.

### 5.2 Tobacco exposure and systemic myeloid priming

Tobacco smoking, the dominant etiological driver of LSCC, is associated with systemic chronic inflammation, including elevated circulating IL-6, IL-8, and VEGF, all established MDSC mobilizing factors (15, 16). Smoking-induced oxidative stress and chronic mucosal inflammation may prime the bone marrow myeloid compartment toward MDSC-biased output, establishing a systemic immunosuppressive background before tumor development. VEGF is overexpressed in LSCC tumor cells in the context of metabolic reprogramming and neoangiogenesis. It simultaneously drives angiogenesis, recruits MDSCs to tumor sites, and blocks their terminal maturation. VEGF therefore forms a mechanistic bridge between the LSCC vascular program and the myeloid immunosuppressive compartment (8).

### 5.3 HPV-negative tumor biology and myeloid-dominant immune exclusion

As an almost uniformly HPV-negative malignancy, LSCC is characterized by lower mutational burden, reduced neoantigen load, and a myeloid-dominant, T cell-excluded microenvironment. This profile contrasts with the T cell-enriched, immunologically active landscape of HPV-positive oropharyngeal carcinoma (16, 19). The clinical consequence of this distinction is that MDSC-mediated suppression of residual antitumor CD8+ T cell activity in LSCC is of proportionally greater consequence. This context suggests that MDSC targeting potentially valuable as a complementary strategy to PD-1 checkpoint inhibition in LSCC.

## 6 MDSC-targeting therapeutic strategies and their rationale for LSCC

Multiple MDSC-targeting agents are in clinical use or advanced development across solid tumors, and these broad strategies are rational for LSCC given the mechanistic framework established above (**Table 1**).

**Table 1:** MDSC-targeting therapeutic strategies: mechanisms, clinical evidence, and rationale for LSCC.

Agent/strategy	MDSC targeting mechanism	Highest clinical evidence level	Key clinical data	LSCC-specific mechanistic rationale	Combination partner	Key reference
All-trans retinoic acid (ATRA)	Promotes MDSC differentiation into mature DCs and macrophages; downregulates ARG1, iNOS, PD-L1; upregulates HLA-DR	Phase I/II (metastatic melanoma)	ORR 71%, CR 50%, mPFS 20.3 months; effectively reduced circulating MDSCs	Directly reverses myeloid maturation block shared with LSCC TAN biology; approved clinical agent with established safety	Anti-PD-1 (pembrolizumab)	Tobin RP et al. (34)
PI3Kγ inhibitor (eganelisib, IPI-549)	Inhibits myeloid-specific PI3Kγ; repolarizes immunosuppressive MDSCs and TAMs; enhances T cell function	Phase 1/1b MARIO-1 (solid tumors including HNSCC)	Acceptable safety; preliminary clinical benefit with nivolumab; pharmacodynamic myeloid remodeling confirmed	PI3K-AKT pathway directly activated by G-CSF/GM-CSF in LSCC TANs; mechanistically direct target at shared signaling node	Anti-PD-1 (nivolumab)	Hong DS et al. (36)
GM-CSF neutralization / G-CSFR blockade	Blocks upstream cytokine drivers of MDSC mobilization from bone marrow and reverses TAN PD-L1 upregulation	Preclinical and *in vitro* evidence in LSCC; clinical trials ongoing in other settings	GM-CSF neutralization reverses PD-L1 on LSCC TANs in experimental models	G-CSF and GM-CSF secretion by LSCC tumor cells is experimentally established; upstream MDSC mobilization blockade	Anti-PD-1	Zhu X et al. (5), Tang D et al. (6)
Anti-VEGF (bevacizumab + ICI)	Reduces MDSC tumor recruitment and differentiation arrest; anti-angiogenic TME remodeling	Phase II real-world data (R/M HNSCC)	Preliminary ORR signals in HNSCC real-world series; MDSC reduction confirmed in preclinical models	VEGF overexpressed in LSCC in context of metabolic reprogramming; dual angiogenic and MDSC-recruiting role	Anti-PD-1/PD-L1	Hua Y et al. (37), Ma K et al. (8)
ATRA + anti-VEGF + ICI (triple combination)	Multi-node targeting: upstream MDSC depletion (anti-VEGF) + differentiation (ATRA) + T cell reactivation (ICI)	Phase II ongoing (MSS colorectal cancer, NCT05999812); concept stage for LSCC	Safety lead-in reported; preclinical rationale strong for MDSC + VEGF dual inhibition with ICI	Mechanistic rationale strongest in LSCC given co-established VEGF, GM-CSF, and G-CSF secretory program	Anti-PD-L1 + anti-VEGF	Ozer M et al. (35)
LOX-1+ PMN-MDSC (liquid biopsy biomarker, not a therapeutic)	Peripheral blood flow cytometry biomarker: quantifies immunosuppressive PMN-MDSC burden before and during ICI therapy	Validated in multicenter cohort of R/M HNSCC (pembrolizumab)	LOX-1+ PMN-MDSCs independently predict ICI resistance and inferior OS/PFS; validated in independent cohort	Non-invasive; directly applicable to LSCC ICI trial design, patient stratification, and on-treatment pharmacodynamic assessment	N/A (biomarker)	Asquino A et al. (18)
CXCR1/2 antagonist (navarixin)	Blocks CXCR1/2-driven transcriptional polarization of PMN-MDSC/TAN toward immunosuppressive, ROS/ARG1-high phenotype	Phase 2 randomized (CRPC, MSS-CRC, NSCLC)	Acceptable safety with pembrolizumab; mechanistic evidence of reduced neutrophil immunosuppressive function	Targets the CXCR4+ TAN-PMN-MDSC continuum described in LSCC	Anti-PD-1 (pembrolizumab)	Armstrong AJ et al. (39), Kwak JW et al. (38)
STAT3 pathway inhibition	Blocks JAK2-STAT3/STAT5 signaling sustaining MDSC survival, ARG1/PD-L1 expression	Preclinical/early-phase, multiple tumor types	Mechanistically validated as PMN-MDSC driver via tumor-intrinsic JAK2/STAT3 axis in HCC	Directly downstream of JAK2-STAT5 node implicated in LSCC MDSC differentiation	Anti-PD-1/PD-L1	Ibrahim A et al. (40), Zhang Z et al. (23)
HDAC inhibitor (entinostat)	Promotes MDSC differentiation/reprogramming, sensitizing myeloid-dominant tumors to ICI	Phase II (metastatic PDAC)	11% ORR, durable responses (median 10.2 months) in chemorefractory, ICI-resistant tumors	Direct analogy to LSCC myeloid-dominant, T cell-excluded TME	Anti-PD-1 (nivolumab)	Baretti M et al. (25)
S100A9 inhibitor (tasquinimod)	Oral inhibitor of S100A9, regulator of MDSC trafficking and function	Phase I/II (prostate cancer, multiple myeloma)	Clinical activity in heavily pretreated, MDSC-rich malignancies; established oral safety	Repurposable oral MDSC-function inhibitor for future LSCC-ICI combination	ICI/IMiD combinations	Vogl DT et al. (41)

ARG1, arginase-1; ATRA, all-trans retinoic acid; CR, complete response; DC, dendritic cell; G-CSF, granulocyte colony-stimulating factor; G-CSFR, granulocyte colony-stimulating factor receptor; GM-CSF, granulocyte-macrophage colony-stimulating factor; HLA-DR, human leukocyte antigen-DR isotype; HNSCC, head and neck squamous cell carcinoma; ICI, immune checkpoint inhibitor; iNOS, inducible nitric oxide synthase; LOX-1, lectin-type oxidized low-density lipoprotein receptor 1; LSCC, laryngeal squamous cell carcinoma; MDSC, myeloid-derived suppressor cell; mPFS, median progression-free survival; MSS, microsatellite stable; ORR, objective response rate; OS, overall survival; PD-1, programmed cell death protein 1; PD-L1, programmed death-ligand 1; PFS, progression-free survival; PI3K-AKT, phosphoinositide 3-kinase/protein kinase B signaling axis; PI3Kγ, phosphoinositide 3-kinase gamma; PMN-MDSC, polymorphonuclear myeloid-derived suppressor cell; R/M, recurrent/metastatic; TAM, tumor-associated macrophage; TAN, tumor-associated neutrophil; TME, tumor microenvironment; VEGF, vascular endothelial growth factor; CXCR1/2, C-X-C chemokine receptor type 1/2; HDAC, histone deacetylase; JAK2, Janus kinase 2; STAT3/STAT5, signal transducer and activator of transcription 3/5; S100A9, S100 calcium-binding protein A9; CRPC, castration-resistant prostate cancer; MSS-CRC, microsatellite-stable colorectal cancer; NSCLC, non-small cell lung cancer; PDAC, pancreatic ductal adenocarcinoma.

### 6.1 All-trans retinoic acid as a differentiation agent

Through nuclear retinoic acid receptor activation, ATRA promotes the differentiation of MDSCs into immunostimulatory mature dendritic cells and macrophages. This process downregulates ARG1, iNOS, TGF-β, and PD-L1 while upregulating HLA-DR and co-stimulatory molecule expression (32, 33). This differentiation mechanism converts immunosuppressive myeloid cells into antigen-presenting ones rather than depleting them, making ATRA particularly compatible with long-term ICI combination therapy. ATRA has a well-characterized clinical safety profile through its approval for acute promyelocytic leukemia. A phase I/II clinical trial combined ATRA with pembrolizumab in anti-PD-1-naive patients with stage IV melanoma. The combination achieved an overall response rate of 71 percent with 50 percent complete responses and a median progression-free survival of 20.3 months, substantially exceeding historical outcomes with pembrolizumab monotherapy. It also reduced circulating MDSC frequency in responders (34). A phase II trial combining ATRA, bevacizumab, and atezolizumab in microsatellite-stable metastatic colorectal cancer was designed specifically on the basis of preclinical evidence that ATRA reduces MDSCs and enhances anti-VEGF and ICI synergy, further supporting the multi-node concept (35). ATRA is among the most readily translatable MDSC-targeting agents for LSCC ICI combinations given its clinical availability and validated MDSC differentiation activity.

### 6.2 PI3Kγ inhibition to repolarize myeloid immunosuppression

PI3Kγ is predominantly expressed in myeloid cells and maintains MDSCs and tumor-associated macrophages in an immunosuppressive state. Eganelisib (IPI-549) is a first-in-class orally administered PI3Kγ inhibitor. It demonstrated antitumor activity both alone and in combination with PD-1 and PD-L1 inhibitors in preclinical models (36). MARIO-1 demonstrated an acceptable safety profile and preliminary clinical benefit for eganelisib combined with nivolumab, with pharmacodynamic evidence of myeloid TME remodeling. The mechanistic relevance to LSCC is direct. The PI3K-AKT pathway activated by G-CSF and GM-CSF in LSCC TANs and bone marrow MDSC progenitors is the same pathway that eganelisib targets. Eganelisib is therefore a mechanism-based intervention in this tumor type.

### 6.3 G-CSF receptor and GM-CSF neutralization

Neutralizing approaches targeting G-CSFR or GM-CSF signaling represent the most LSCC-specific MDSC-targeting strategy proposed here. GM-CSF neutralization in LSCC models reversed PD-L1 upregulation on tumor-infiltrating neutrophils and restored T cell-mediated tumor cell clearance (6). Applied systemically, the same neutralization would interrupt the upstream mobilization of MDSC progenitors from the bone marrow through the shared GM-CSF/PI3K-AKT pathway, targeting the apex of the myeloid immunosuppressive cascade in LSCC. This strategy is distinct from downstream MDSC differentiation or depletion approaches and provides the most mechanistically proximal intervention in the LSCC-specific cytokine-driven myeloid suppression program.

### 6.4 Anti-VEGF combination approaches

Anti-VEGF agents including bevacizumab reduce MDSC accumulation in preclinical tumor models and have shown preliminary activity when combined with anti-PD-1 therapy in patients with recurrent and metastatic HNSCC in real-world clinical analyses (37). Metabolic reprogramming that sustains VEGF secretion in LSCC provides a direct rationale for the anti-VEGF plus ICI combination in this disease (8, 37).

We propose that circulating LOX-1+ PMN-MDSCs, measurable by standard peripheral blood flow cytometry without invasive tumor sampling, should be incorporated as a mandatory stratification biomarker and pharmacodynamic endpoint in all prospective LSCC ICI clinical trials. As established in the HNSCC ICI resistance literature, baseline LOX-1+ PMN-MDSC levels predict ICI outcomes and on-treatment dynamics reflect myeloid TME remodeling (18), providing an accessible, non-invasive biomarker axis for adaptive trial design and optimization of patient selection in LSCC.

### 6.5 CXCR1/2 chemokine receptor antagonism

CXCR1/2 blockade (navarixin, SX-682, AZD5069, reparixin) targets the chemokine receptors that direct PMN-MDSC and TAN trafficking and functional polarization. Recent mechanistic work demonstrates that CXCR1/2 antagonism acts primarily by blocking neutrophil/PMN-MDSC transcriptional polarization toward an immunosuppressive, reactive-oxygen-species- and ARG1-high phenotype, rather than simply preventing trafficking into the tumor. This mechanism explains why these agents remain active without inducing infectious complications despite persistent neutrophil infiltration (38). A phase 2 randomized trial of the CXCR2 antagonist navarixin combined with pembrolizumab in castration-resistant prostate cancer, microsatellite-stable colorectal cancer, and non-small cell lung cancer demonstrated an acceptable safety profile (39). Because LSCC-infiltrating TANs are generated downstream of the same G-CSF/GM-CSF axis and display CXCR4+, aged, immunosuppressive phenotypes, CXCR1/2 antagonism represents a direct pharmacological approach to the myeloid population implicated mechanistically in this Perspective.

### 6.6 STAT3 pathway inhibition

G-CSF and GM-CSF signal in part through JAK2-STAT3/STAT5 to sustain MDSC survival, ARG1 and PD-L1 expression, and differentiation arrest. STAT3-targeted strategies have been comprehensively cataloged as an “MDSC checkpoint” amenable to combination immunotherapy (40), and a tumor-intrinsic transcription factor driving JAK2/STAT3 activation has been directly shown to be responsible for PMN-MDSC-mediated checkpoint resistance in hepatocellular carcinoma (23). Because JAK2-STAT5 signaling downstream of GM-CSF is already implicated in bone marrow MDSC differentiation in our proposed LSCC model, STAT3/STAT5 pathway inhibition constitutes a mechanistically direct, rather than indirect, intervention.

### 6.7 HDAC inhibition as an MDSC-reprogramming strategy

Histone deacetylase inhibitors reduce myeloid-mediated immunosuppression and promote MDSC differentiation. In a phase II trial, entinostat combined with nivolumab in patients with checkpoint-refractory, myeloid-dominant metastatic pancreatic ductal adenocarcinoma produced durable partial responses in a treatment-refractory population (11% objective response rate, median response duration 10.2 months) (25), providing direct proof-of-concept that pharmacologically reprogramming myeloid immunosuppression can convert an ICI-refractory, myeloid-dominant tumor into a responsive one. HDAC-inhibitor efficacy on MDSCs is context-dependent across tumor types, underscoring the need for LSCC-specific pharmacodynamic validation before clinical application.

### 6.8 S100A9 inhibition (tasquinimod)

Tasquinimod is an oral small-molecule inhibitor of S100A9, a key surface regulator of MDSC trafficking, differentiation arrest, and immunosuppressive function. Originally developed for castration-resistant prostate cancer, tasquinimod has recently shown clinical activity, including objective responses, in heavily pretreated patients with multiple myeloma (a malignancy characterized by S100A9-driven, MDSC-rich bone marrow immunosuppression) (41). As a first-in-class oral MDSC-function inhibitor with an established tolerability profile across two malignancies, tasquinimod represents an additional repurposing candidate for LSCC-ICI combination once dedicated LSCC MDSC characterization has been completed.

## 7 Discussion

MDSCs represent a plausible and treatable immunosuppressive node in LSCC that has been overlooked by the research community. Several independent lines of evidence support this position. The LSCC tumor secretome is enriched in G-CSF, GM-CSF, VEGF, and IL-6, all established MDSC mobilizing and differentiation factors (5, 6, 8, 16). The PI3K-AKT signaling axis activated by G-CSF and GM-CSF in LSCC TANs is mechanistically shared with bone marrow MDSC differentiation programs (5, 20). The transcriptional landscape of circulating PMN-MDSCs and TANs overlaps at the single-cell level across multiple cancer types, supporting a biological continuum between these myeloid populations (21, 22). The predominantly elderly, tobacco-exposed, HPV-negative demographic profile of LSCC patients brings together systemic factors that amplify MDSC accumulation through SASP signaling, myeloid-biased hematopoiesis, and immune exclusion (26–28). Finally, the HNSCC literature has validated LOX-1+ PMN-MDSCs as predictors of ICI resistance, providing an immediately applicable biomarker framework for LSCC clinical trials (18).

The translational implications are concrete and testable. Current predictive biomarker strategies for LSCC ICI therapy rely principally on PD-L1 combined positive score, tumor mutational burden, and tumor-infiltrating lymphocyte density, none of which capture the myeloid immunosuppressive contribution to ICI resistance (1, 16). Incorporating circulating MDSC quantification into LSCC biomarker panels would add a modifiable, pharmacodynamically trackable dimension to these predictive frameworks. ATRA and eganelisib carry established clinical safety profiles and have demonstrated MDSC-targeting efficacy in ICI combination contexts (34, 36), lowering the barrier to early-phase clinical investigation of these agents in LSCC.

We acknowledge important limitations qualifying this Perspective. The mechanistic model connecting the LSCC tumor secretome to MDSC expansion rests on converging indirect evidence rather than direct experimental data in LSCC patient samples or LSCC-specific *in vivo* models. No LSCC-specific MDSC characterization study currently exists, and this is the primary scientific gap requiring experimental resolution. Closing this gap requires three complementary experimental approaches. The first approach, ex vivo functional validation, would analyze fresh LSCC surgical specimens and matched peripheral blood should be analyzed by flow cytometry for CD11b+CD14-CD15+LOX-1+ PMN-MDSCs and CD11b+CD14+HLA-DR-CD15- M-MDSCs, followed by standardized autologous T cell suppression co-culture assays performed strictly according to international consensus criteria (9), to determine whether LSCC-infiltrating and circulating myeloid populations meet the functional definition of MDSCs rather than merely overlapping phenotypically with TANs. The second approach, mechanistic causality testing, would co-culture LSCC patient-derived organoids or air-liquid-interface tumor explant cultures, an approach now validated for reconstructing the head and neck tumor-immune microenvironment ex vivo (42), could be co-cultured with autologous myeloid progenitors under G-CSF/GM-CSF neutralization or PI3K-AKT inhibition, modeled on combined genetic perturbation and functional suppression assay recently used to establish causal PMN-MDSC-driven checkpoint resistance in hepatocellular carcinoma (23), to test whether disrupting this cytokine axis directly attenuates MDSC-mediated T cell suppression. The third approach, *in vivo* modeling, would subject syngeneic or humanized-mouse LSCC models subjected to G-CSF/GM-CSF neutralization, PI3Kγ inhibition, or CXCR1/2 antagonism (38, 39), with paired assessment of tumor-infiltrating myeloid populations, anti-PD-1 response, and survival, would establish whether this axis is causally necessary for ICI resistance in LSCC rather than merely correlated with it.

The definitional boundary between PMN-MDSCs and immunosuppressive TANs in LSCC must be resolved through standardized functional suppression assays applied to LSCC samples alongside rigorous consensus phenotyping, as the international community has established (9). MDSC subset heterogeneity and the absence of universally specific surface markers have historically complicated cross-study integration in HNSCC and will similarly challenge LSCC MDSC research unless rigorous standardization is adopted from the outset.

The LSCC immunotherapy field is in transition, with perioperative ICI regimens being explored for laryngeal function preservation and novel bispecific antibodies and antibody-drug conjugates entering early-phase evaluation (1, 43). These approaches are unlikely to reach their full potential without overcoming the myeloid immunosuppressive barrier that characterizes this disease. Translating this framework into practice requires a staged, falsifiable evidentiary program rather than a direct leap to large interventional trials. We propose four sequential tiers, each gated by an explicit go/no-go criterion. Tier 1 (retrospective/correlative) would apply multiplex immunohistochemistry and peripheral blood flow cytometry for LOX-1+ PMN-MDSCs, ARG1, and PD-L1 to archived LSCC-only cohorts with known ICI outcomes, testing whether the HNSCC-pooled biomarker association replicates when LSCC is analyzed as an independent entity. A significant association would justify Tier 2. Tier 2 (prospective biomarker-embedded studies) would incorporate baseline and on-treatment LOX-1+ PMN-MDSC measurement as a mandatory exploratory endpoint within already-planned or recruiting LSCC ICI trials, without altering treatment allocation. Confirmation of predictive and pharmacodynamic value would justify Tier 3. Tier 3 (mechanistic causality) would employ the ex vivo functional suppression assays, patient-derived organoid/explant co-culture, and *in vivo* cytokine-neutralization models detailed above. Concordant causal evidence would justify Tier 4. Tier 4 (early-phase interventional trials) would then test repurposed MDSC-targeting agents (ATRA, entinostat, or CXCR1/2 antagonists) with established safety profiles combined with anti-PD-1 blockade under biomarker-adaptive design. This tiered structure specifies precisely when, and under what evidentiary conditions, escalation to larger clinical studies or novel drug design becomes justified, rather than presenting an untethered hypothesis.

## Data Availability

The original contributions presented in the study are included in the article/supplementary material. Further inquiries can be directed to the corresponding authors.

## References

[1] Fuereder T Kocher F Vermorken JB . Systemic therapy for laryngeal carcinoma. Front Oncol. (2025) 15:1541385. doi: 10.3389/fonc.2025.1541385 40104492 PMC11913863

[2] Burtness B Harrington KJ Greil R Soulières D Tahara M de Castro GJ . Pembrolizumab alone or with chemotherapy versus cetuximab with chemotherapy for recurrent or metastatic squamous cell carcinoma of the head and neck (KEYNOTE-048): a randomised, open-label, phase 3 study. Lancet (London England). (2019) 394:1915–28. doi: 10.1016/S0140-6736(19)32591-7 31679945

[3] Tahara M Greil R Rischin D Harrington KJ Burtness B de Castro G . Pembrolizumab with or without chemotherapy in recurrent or metastatic head and neck squamous cell carcinoma: 5-year follow-up from the randomized phase III KEYNOTE-048 study. Eur J Cancer (Oxford Engl 1990). (2025) 221:115395. doi: 10.1016/j.ejca.2025.115395 40262400

[4] Sun Y Chen S Lu Y Xu Z Fu W Yan W . Single-cell transcriptomic analyses of tumor microenvironment and molecular reprograming landscape of metastatic laryngeal squamous cell carcinoma. Commun Biol. (2024) 7:63. doi: 10.1038/s42003-024-05765-x 38191598 PMC10774275

[5] Zhu X Heng Y Ma J Zhang D Tang D Ji Y . Prolonged survival of neutrophils induced by tumor-derived G-CSF/GM-CSF promotes immunosuppression and progression in laryngeal squamous cell carcinoma. Advanced Sci (Weinheim Baden-Württemberg Germany). (2024) 11:e2400836. doi: 10.1002/advs.202400836 39447112 PMC11633501

[6] Tang D Zhang D Heng Y Zhu X Lin H Zhou J . Tumor-infiltrating PD-L1+ neutrophils induced by GM-CSF suppress T cell function in laryngeal squamous cell carcinoma and predict unfavorable prognosis. J Inflammation Res. (2022) 15:1079–97. doi: 10.2147/JIR.S347777 35210813 PMC8859980

[7] Xu L Li W Liu D Cao J Ge J Liu X . ANXA3-rich exosomes derived from tumor-associated macrophages regulate ferroptosis and lymphatic metastasis of laryngeal squamous cell carcinoma. Cancer Immunol Res. (2024) 12:614–30. doi: 10.1158/2326-6066.CIR-23-0595 38393971

[8] Ma K Mao Q Fei B Ni T Zhang Z Ni H . Metabolic reprogramming and immune microenvironment characteristics in laryngeal carcinoma: advances in immunotherapy. Front Immunol. (2025) 16:1589243. doi: 10.3389/fimmu.2025.1589243 40370437 PMC12075248

[9] Bronte V Brandau S Chen S Colombo MP Frey AB Greten TF . Recommendations for myeloid-derived suppressor cell nomenclature and characterization standards. Nat Commun. (2016) 7:12150. doi: 10.1038/ncomms12150 27381735 PMC4935811

[10] Gabrilovich DI Nagaraj S . Myeloid-derived suppressor cells as regulators of the immune system. Nat Rev Immunol. (2009) 9:162–74. doi: 10.1038/nri2506 19197294 PMC2828349

[11] Jiang W Hu K Liu X Gao J Zhu L . Single-cell transcriptome analysis reveals the clinical implications of myeloid-derived suppressor cells in head and neck squamous cell carcinoma. Pathol Oncol Res POR. (2023) 29:1611210. doi: 10.3389/pore.2023.1611210 37475874 PMC10354270

[12] Zhao X Zhu Y He Y Gu W Zhou Q Jin B . Unraveling the immune evasion mechanisms in the tumor microenvironment of head and neck squamous cell carcinoma. Front Immunol. (2025) 16:1597202. doi: 10.3389/fimmu.2025.1597202 40438103 PMC12116449

[13] Wang H Zhou F Qin W Yang Y Li X Liu R . Metabolic regulation of myeloid-derived suppressor cells in tumor immune microenvironment: targets and therapeutic strategies. Theranostics. (2025) 15:2159–84. doi: 10.7150/thno.105276 39990210 PMC11840731

[14] Lu J Luo Y Rao D Wang T Lei Z Chen X . Myeloid-derived suppressor cells in cancer: therapeutic targets to overcome tumor immune evasion. Exp Hematol Oncol. (2024) 13:39. doi: 10.1186/s40164-024-00505-7 38609997 PMC11010322

[15] Ruffin AT Li H Vujanovic L Zandberg DP Ferris RL Bruno TC . Improving head and neck cancer therapies by immunomodulation of the tumour microenvironment. Nat Rev Cancer. (2023) 23:173–88. doi: 10.1038/s41568-022-00531-9 36456755 PMC9992112

[16] Cillo AR Kürten CHL Tabib T Qi Z Onkar S Wang T . Immune landscape of viral- and carcinogen-driven head and neck cancer. Immunity. (2020) 52:183–99. doi: 10.1016/j.immuni.2019.11.014 31924475 PMC7201194

[17] Silver NL Dai J Kerr TD Altemus J Garg R Simmons H . Intratumoral bacteria are immunosuppressive and promote immunotherapy resistance in head and neck squamous cell carcinoma. Nat Cancer. (2026) 7:80–97. doi: 10.1038/s43018-025-01067-1 41482523 PMC12823012

[18] Asquino A Cirillo A Strigari L Pace A Napoletano C Tuosto L . Circulating CD137+ Treg cells and LOX-1+ PMN-MDSCs as biomarkers of immunotherapy resistance in (R/M) HNSCC patients. J Exp Clin Cancer Res CR. (2025) 44:316. doi: 10.1186/s13046-025-03574-6 41340154 PMC12676864

[19] Tosi A Parisatto B Menegaldo A Spinato G Guido M Del Mistro A . The immune microenvironment of HPV-positive and HPV-negative oropharyngeal squamous cell carcinoma: a multiparametric quantitative and spatial analysis unveils a rationale to target treatment-naive tumors with immune checkpoint inhibitors. J Exp Clin Canc Res. (2022) 41:279. doi: 10.1186/s13046-022-02481-4 36123711 PMC9487049

[20] He K Liu X Hoffman RD Shi R Lv G Gao J . G-CSF/GM-CSF-induced hematopoietic dysregulation in the progression of solid tumors. FEBS Open Bio. (2022) 12:1268–85. doi: 10.1002/2211-5463.13445 35612789 PMC9249339

[21] Pettinella F Mariotti B Lattanzi C Bruderek K Donini M Costa S . Surface CD52, CD84, and PTGER2 mark mature PMN-MDSCs from cancer patients and G-CSF-treated donors. Cell Rep Med. (2024) 5:101380. doi: 10.1016/j.xcrm.2023.101380 38242120 PMC10897522

[22] Lattanzi C Bianchetto-Aguilera F Donini M Pettinella F Caveggion E Castellucci M . Uncovering common transcriptional features shared by mature peripheral blood PMN-MDSCs and tumor-infiltrating neutrophils in humans. Oncoimmunology. (2025) 14:2521396. doi: 10.1080/2162402X.2025.2521396 40590753 PMC12218443

[23] Zhang Z Huang W Hu D Jiang J Zhang J Wu Z . E-twenty-six-specific sequence variant 5 (ETV5) facilitates hepatocellular carcinoma progression and metastasis through enhancing polymorphonuclear myeloid-derived suppressor cell (PMN-MDSC)-mediated immunosuppression. Gut. (2025) 74:1137–49. doi: 10.1136/gutjnl-2024-333944 40015948

[24] Oh JM Kim S Tsung C Kent E Jain A Ruff SM . Comprehensive review of the resistance mechanisms of colorectal cancer classified by therapy type. Front Immunol. (2025) 16:1571731. doi: 10.3389/fimmu.2025.1571731 40777019 PMC12328380

[25] Baretti M Danilova L Durham JN Betts CB Cope L Sidiropoulos DN . Entinostat in combination with nivolumab in metastatic pancreatic ductal adenocarcinoma: a phase 2 clinical trial. Nat Commun. (2024) 15:9801. doi: 10.1038/s41467-024-52528-7 39532835 PMC11557583

[26] Talarico G Orecchioni S Falvo P Bertolini F . Myeloid-derived suppressor cells (MDSCs) at the crossroad of senescence and cancer. Cancers. (2025) 17:2251. doi: 10.3390/cancers17132251 40647547 PMC12248894

[27] Keltsch E Greiner J Wahl L Knape I Tews D Denkinger M . Aging modulates the immunosuppressive, polarizing and metabolic functions of blood-derived myeloid-derived suppressor cells (MDSCs). Immun Ageing I A. (2025) 22:29. doi: 10.1186/s12979-025-00524-w 40629365 PMC12235767

[28] Fane M Weeraratna AT . How the ageing microenvironment influences tumour progression. Nat Rev Cancer. (2020) 20:89–106. doi: 10.1038/s41568-019-0222-9 31836838 PMC7377404

[29] Veglia F Perego M Gabrilovich D . Myeloid-derived suppressor cells coming of age. Nat Immunol. (2018) 19:108–19. doi: 10.1038/s41590-017-0022-x 29348500 PMC5854158

[30] Su Y Wu R Ai H Zhong Z Zou L Wang Z . Defining the marker and developmental trajectory of myeloid-derived suppressor cells in aging by single-cell transcriptomics. NPJ Aging. (2025) 12:18. doi: 10.1038/s41514-025-00317-x 41444228 PMC12855942

[31] Liu N Wu J Deng E Zhong J Wei B Cai T . Immunotherapy and senolytics in head and neck squamous cell carcinoma: phase 2 trial results. Nat Med. (2025) 31:3047–61. doi: 10.1038/s41591-025-03873-7 40855191

[32] Gao X Sui H Zhao S Gao X Su Y Qu P . Immunotherapy targeting myeloid-derived suppressor cells (MDSCs) in tumor microenvironment. Front Immunol. (2021) 11:585214. doi: 10.3389/fimmu.2020.585214 33613512 PMC7889583

[33] He S Zheng L Qi C . Myeloid-derived suppressor cells (MDSCs) in the tumor microenvironment and their targeting in cancer therapy. Mol Cancer. (2025) 24:5. doi: 10.1186/s12943-024-02208-3 39780248 PMC11707952

[34] Tobin RP Cogswell DT Cates VM Davis DM Borgers JSW Van Gulick RJ . Targeting MDSC differentiation using ATRA: a phase I/II clinical trial combining pembrolizumab and all-trans retinoic acid for metastatic melanoma. Clin Cancer Res Off J Am Assoc For Cancer Res. (2023) 29:1209–19. doi: 10.1158/1078-0432.CCR-22-2495 36378549 PMC10073240

[35] Ozer M Verma N Brown TJ Hsieh D Chung V Al Mutar S . Safety and tolerability of all-trans-retinoic acid, bevacizumab, and atezolizumab in refractory microsatellite stable colorectal cancer. J Clin Oncol. (2026) 44:114. doi: 10.1200/JCO.2026.44.2_suppl.114

[36] Hong DS Postow M Chmielowski B Sullivan R Patnaik A Cohen EEW . Eganelisib, a first-in-class PI3Kγ inhibitor, in patients with advanced solid tumors: results of the phase 1/1b MARIO-1 trial. Clin Cancer Res Off J Am Assoc For Cancer Res. (2023) 29:2210–9. doi: 10.1158/1078-0432.CCR-22-3313 37000164 PMC10388696

[37] Hua Y Dong R Jin T Jin Q Chen X . Anti-PD-1 monoclonal antibody combined with anti-VEGF agent is safe and effective in patients with recurrent/metastatic head and neck squamous cancer as second-line or beyond treatment. Front Oncol. (2022) 12:781348. doi: 10.3389/fonc.2022.781348 35280787 PMC8908371

[38] Kwak JW Nguyen HQ Camai A Huffman GM Mekvanich S Kenney NN . CXCR1/2 antagonism inhibits neutrophil function and not recruitment in cancer. Oncoimmunology. (2024) 13:2384674. doi: 10.1080/2162402X.2024.2384674 39076249 PMC11285219

[39] Armstrong AJ Geva R Chung HC Lemech C Miller WHJ Hansen AR . CXCR2 antagonist navarixin in combination with pembrolizumab in select advanced solid tumors: a phase 2 randomized trial. Invest New Drug. (2024) 42:145–59. doi: 10.1007/s10637-023-01410-2 38324085 PMC11076327

[40] Ibrahim A Mohamady Farouk Abdalsalam N Liang Z Kashaf Tariq H Li R O Afolabi L . MDSC checkpoint blockade therapy: a new breakthrough point overcoming immunosuppression in cancer immunotherapy. Cancer Gene Ther. (2025) 32:371–92. doi: 10.1038/s41417-025-00886-9 40140724 PMC11976280

[41] Vogl DT Nefedova Y Wileyto EP Whittington S Diaczynsky C Nguyen CT . Clinical activity of novel targeting of S100A9 with tasquinimod for relapsed and refractory multiple myeloma (RRMM). J Clin Oncol. (2025) 43:7555. doi: 10.1200/JCO.2025.43.16_suppl.7555 42148471

[42] Zheng L Tang W Guo S Chen L Wang S Liu F . Reconstituting the head and neck tumor microenvironment with air-liquid interface organoids. Front Oncol. (2026) 15:1736505. doi: 10.3389/fonc.2025.1736505 41551162 PMC12807938

[43] Fang Q Li X Xu P Cao F Wu D Zhang X . PD-1 inhibitor combined with paclitaxel and cisplatin in the treatment of recurrent and metastatic hypopharyngeal/laryngeal squamous cell carcinoma: efficacy and survival outcomes. Front Immunol. (2024) 15:1353435. doi: 10.3389/fimmu.2024.1353435 38827739 PMC11140072

